# Digital Control and Demodulation Algorithm for Compact Open-Loop Fiber-Optic Gyroscope

**DOI:** 10.3390/s23031473

**Published:** 2023-01-28

**Authors:** Lin Chen, Zhao Huang, Yuzheng Mao, Biqiang Jiang, Jianlin Zhao

**Affiliations:** 1 Key Laboratory of Light Field Manipulation and Information Acquisition, Ministry of Industry and Information Technology, Shaanxi Key Laboratory of Optical Information Technology, School of Physical Science and Technology, Northwestern Polytechnical University, Xi’an 710129, China; 2 Xi’an Flight Automatic Control Research Institute, Xi’an 710065, China

**Keywords:** open-loop fiber-optic gyroscope, digital control algorithm, compact, cost-effective

## Abstract

With the advantages of small size, low cost, and moderate accuracy, an open-loop fiber-optic gyroscope (FOG) has a wide range of applications around control and automation. For the most cost-sensitive applications, a simple and stable digital algorithm with a reduced control-circuit volume and cost is highly desirable to realize high-precision control of a FOG. In this work, a new algorithm for an open-loop FOG is proposed based on the discrete multi-point demodulation in the sinusoidal modulation period. Utilizing this algorithm, stable control and angular velocity calculation of a gyro are realized with effectively suppressed gyro error. The use of this algorithm greatly reduces the requirements for processing power and simplifies the gyro circuit. Based on this algorithm, a digital FOG with a volume of only 25 × 20 × 40 mm^3^ achieves a bias instability of less than 0.15°/h, an angle random walk (*ARW*) of less than 0.015°/√h, a start-up time of less than 1 s, and a 3 dB bandwidth beyond 160 Hz. This low-cost, compact, and high-performance gyro is sufficient to satisfy the requirements of applications in the navigation and control fields such as unmanned driving.

## 1. Introduction

A fiber-optic gyroscope (FOG) is the core sensor for the navigation, positioning, and attitude control of moving objects [1,2,3]. According to the difference in the control algorithm, it can be divided into closed-loop and open-loop fiber-optic gyroscopes [4]. Although the accuracy of an open-loop fiber-optic gyroscope is not as good as that of a closed-loop one, it consists of an all-fiber architecture without expensive integrated optical modulators, featuring a small size, low cost, and moderate accuracy. Open-loop gyroscopes have drawn much attention in high-end control fields such as automatic driving and robot control [5]. Much research has been carried out on the optical components, electric circuits, and algorithms to further drive down the cost and size of open-loop fiber-optic gyroscopes, the result of which makes them more competitive than Micro Electromechanical System gyro (MEMS) and low-end closed-loop gyros in the market [6,7,8,9]. In terms of hardware circuits and control algorithms, the current research focuses on the realization of a more stable and concise control algorithm to reduce the computational power requirements of the processor. Therefore, it becomes possible to use a simpler hardware configuration to achieve high-precision control of a gyro, thereby reducing the control circuit footprint and cost [10,11,12].

The conventional open-loop gyroscope algorithm demodulates synchronously the amplitudes of the first, second, and fourth harmonic components of its temporal interference waveform, which requires complex multiple synchronous phase-locked solutions and a powerful convolution processor with a high cost and large footprint [13]. In order to overcome this problem, some researchers have produced a solution of characteristic points in the modulation period [12], which can simplify the algorithm. However, this method suffers from a low signal-to-noise ratio. In this work, a control demodulation scheme based on discrete multi-point demodulation is proposed to obtain a high signal-to-noise ratio without complicated multiple synchronous phase-locking. This algorithm allows the implementation of a gyro’s sampling control, signal demodulation, and error suppression into a compact, low-cost FPGA, avoiding the combination of an FPGA and a digital signal processor (DSP) as in a traditional FOG. The size of our open-loop FOG with this algorithm is only 25 × 20 × 40 mm^3^, which is about 50% of the size of similar gyros, e.g., the DSP17XX series from KVH Inc. with a dimension of ϕ45.7 × 22.9 mm^2^. At the same time, the use of a low-arithmetic-power processor significantly reduces the cost of our gyro. This kind of cost-effective and compact gyro provides high performances in bias instability of less than 0.15°/h, angle random walk (*ARW*) of less than 0.015°/√h, start-up time of less than 1 s, and 3 dB bandwidth of more than 160 Hz. These characteristics promise that the open-loop FOG with our new demodulation algorithm can find potential applications in many fields, such as automation and control.

## 2. Demodulation and Control Algorithm

### 2.1. Demodulation Algorithm

**Figure 1:**
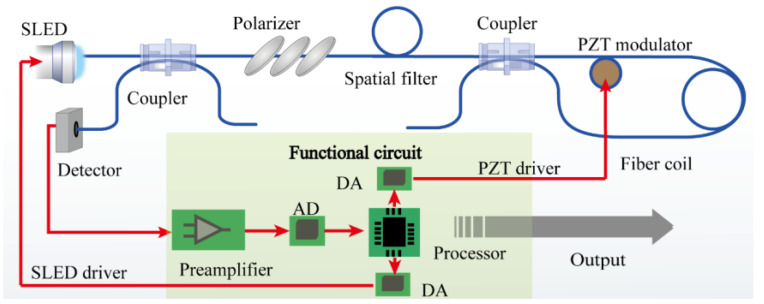
Scheme layout of an open-loop FOG.

The open-loop optical fiber gyroscope constructed in this work is based on the minimization structure of all fiber-optic gyroscopes, as shown in Figure 1. The light from the broad-spectrum light source (SLED module) passing through the coupler enters the polarizer and spatial filter to ensure the purity of the coherent polarization mode and is divided into two counterpropagating beams in the second coupler. Based on the Sagnac effect, the optical path difference *φ*_s_ of the two counterpropagating beams in the fiber coil is proportional to the rotational angular velocity Ω [14],

(1) $$\varphi_{s}=\frac{2\pi LD}{\lambda c}\Omega ,$$

where *L* is the optical fiber length, *D* is the diameter of the fiber coil, *λ* is the central wavelength, and *c* is the speed of light in vacuum. The returned light is filtered again via the polarizer and then enters the detector. The detected interference light intensity is amplified, sampled, and demodulated via the functional circuit to obtain the Sagnac optical path difference.

A modulator is often used to improve the sensitivity of Sagnac phase-shift demodulation. In comparison with a closed-loop FOG, an open-loop FOG has a great cost advantage due to the use of a lead zirconate titanate piezoelectric ceramics (PZT) modulator, which is only 1/100 the price of the lithium niobate electro-optic modulator used in a closed-loop FOG [15]. Compared with the high-frequency square-wave modulation scheme viable with the use of electro-optical modulators, the bandwidth-limited PZT modulator can only use a low-frequency sinusoidal modulation scheme, which places higher demands on the demodulation accuracy [16].

Under the sinusoidal modulation scheme, the interference intensity is given by

(2) $$I(\omega t)=I_{0}+I_{0}\cos(\varphi_{\mathrm{sq}}+\phi_{m}\sin(\omega t+\tau )),$$

where *I*_0_ is half of the maximum light intensity detected and *w* is the angular frequency of modulation. In order to use a low voltage to excite its phase-modulating signal, the PZT is driven close to its resonance frequency. The modulator can only operate efficiently in a small frequency bandwidth of about 5 kHz around its resonance and driving frequencies deviating from the resonance will result in a sharp decay in the PZT oscillation amplitude. Therefore, the effect of multiple harmonics of the PZT is negligible. *τ* represents the phase delay, which corresponds to the sampling delay for this digital gyro, *ϕ*_m_ is the amplitude of phase modulation, and *φ*_sq_ = *φ*_s_ + *φ*_q_, where *φ*_s_ is the Sagnac phase shift and *φ*_q_ represents the polarization error that cannot be decoupled from the Sagnac phase shift and can only be suppressed with optimized optical devices [17,18].

**Figure 2:**
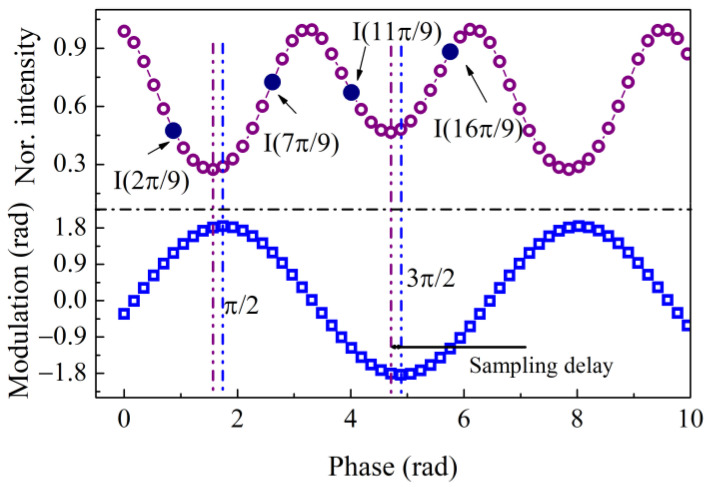
Modulation and discrete sampling of the normalized interference signal.

The interference intensity is converted into a voltage signal with a photo detector followed by a linear preamplifier circuit, as shown in Figure 1, and the amplified voltage signal is sampled evenly with a set sampling frequency, as shown in Figure 2, which is 18 *w/*π in this work to balance the accuracy and hardware resource requirements. Hence, the sampled discrete signals in a modulation cycle are given by

(3) $$I^{\prime }(n)=I_{0}+I_{0}\cos(\varphi_{\mathrm{sq}}+\phi_{m}\sin((n\pi /18)+\tau )),n=0,1,2\ldots 35$$

Define that

(4) $$U=\sum_{n=0}^{35}\left(I^{\prime }(n)\sin(n\pi /18)\right).$$

Since *τ* represents the sampling phase delay for the digital gyro, which is closed-loop controlled to a very small residual amount compared with the modulated phase, by substituting Equation (3) into Equation (4) and making a first-order approximation for terms containing *τ*, we obtain

(5) $$\begin{array}{l} U=\sum_{n=0}^{35}\left(I_{0}\sin(n\pi /18)\right)+\sum_{n=0}^{35}\left(I_{0}\cos(\varphi_{\mathrm{sq}}+\phi_{m}\sin(n\pi /18)+\tau ))\sin(n\pi /18)\right)\\ =\sum_{n=0}^{35}\left((I_{0}\cos\varphi_{\mathrm{sq}}\cos(\phi_{m}\sin(n\pi /18+\tau )\sin(n\pi /18)-I_{0}\sin\varphi_{\mathrm{sq}}\sin(\phi_{m}\sin(n\pi /18+\tau ))\sin(n\pi /18)\right) \end{array}.$$

By using Bessel function expansion, Equation (5) can be written as

(6) $$\begin{array}{l} U\approx \sum_{n=0}^{35}I_{0}\cos\varphi_{\mathrm{sq}}\sin(n\pi /18)(J_{0}(\phi_{m})+2\sum_{k=0}^{\infty }J_{2k}(\phi_{m})\cos2k(n\pi /18))(\cos(\phi_{m}\cos(n\pi /18)\tau )\\ -\sum_{n=0}^{35}2I_{0}\sin\varphi_{\mathrm{sq}}\sin(n\pi /18)\sum_{k=0}^{\infty }J_{2k+1}(\phi_{m})\sin((2k+1)(n\pi /18))(\cos(\phi_{m}\cos(n\pi /18)\tau ) \end{array},$$

where *J* is the Bessel function of the first kind. By making use of

(7) $$\sum_{n=0}^{35}\sin(n\pi /18)\sin((2k+1)(n\pi /18))\approx 0,k\neq 0,$$

and

(8) $$\sum_{n=0}^{35}\sin(n\pi /18)\cos(2k(n\pi /18))\approx 0,$$

and retaining the cumulative non-zero terms, we can obtain

(9) $$U\approx -36I_{0}J_{1}(\phi_{m})\cos^{2}\left(\phi_{m}\tau /2\right)\sin\varphi_{\mathrm{sq}}.$$

Therefore,

(10) $$\varphi_{\mathrm{sq}}=-\arcsin\left(\frac{U}{36I_{0}J_{1}(\phi_{m})\cos^{2}(\phi_{m}\tau /2)}\right).$$

From Equations (7) and (8), the main deviation in the derivation of the angular rate comes from the higher harmonic residuals generated by the inner product of the sinusoidal signal generated at the discrete point and the interferometric signal. This error can lead to deviation in the scale factor and nonlinearity in the gyro output, the former of which can be corrected via rate calibration and compensation. Meanwhile, the nonlinear error in the Sagnac phase shift can be suppressed by adding single-cycle sampling points. The sampling rate of 36 points per cycle used in this work is a compromise between the acceptable gyro output accuracy and the limited hardware resources. Nevertheless, our numerical simulation results suggest that the nonlinear error of the gyro can be suppressed with more sampling points per cycle, and linearity within 100 ppm is achievable.

As a result, stable power *I*_0_, settled modulation amplitude *ϕ*_m_, and smallest possible sampling delay *τ* are all necessary to precisely obtain the Sagnac phase shift. The control algorithm is described as follows.

### 2.2. Control of the Synchronous Sampling Delay

In the algorithm, the processor sends commands to the DA to drive the PZT to generate a sinusoidal modulated signal, which is shown in the lower part of Figure 2. The phase modulation is loaded on the interferometric light intensity, and the normalized light intensity is shown in the upper part of Figure 2. The sampling signal is synchronized with the drive signal, but the interference signal lags behind this original sampling signal due to the capacitance of the piezoelectric ceramic and the delay of the processing circuit. In the algorithm, the sampling delay should be precisely controlled to achieve accurate demodulation, and the methods are as follows.

Equally spaced sampling points are determined around the points π/2 and 3π/2, as shown in Figure 2. With a picking interval *ξ*, we can determine that

(11) $$\Delta =I(\pi /2+\xi )-I(\pi /2-\xi )+I(3\pi /2+\xi )-I(\pi /2-\xi )=4I_{0}\tau \sin\xi \cos\varphi_{\mathrm{sq}}\sin(\phi_{m}\cos\xi ).$$

**Figure 3:**
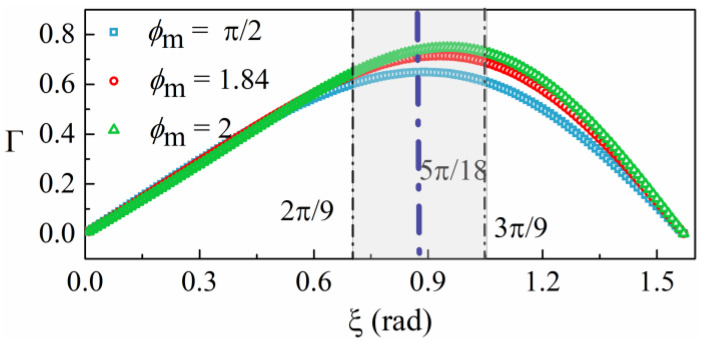
Optimized picking interval for the elimination of the synchronous sampling delay, *τ*, at different modulation amplitudes.

Since the values of *φ*_sq_ and *ξ* range from −π/2 to π/2, Δ = 0 only happens when *τ* = 0. Therefore, the synchronous sampling delay *τ* could be controlled with feedback to make *τ* = 0 by measuring Δ. Choosing the appropriate picking interval *ξ* can maximize Γ = sin *ξ* sin (*ϕ*_m_ cos *ξ*) so as to obtain an optimum sensitivity of feedback control for Δ. With different values of *ϕ*_m_, the variation in Γ as a function of the picking interval is shown in Figure 3. Note that the optimum *ξ* is between 0.8 and 1, and considering the discreteness of sampling, *ξ* is set to a value of 5π/18. Thus, *τ* can be controlled with calculations of the 4 sampling points at 2π/9, 7π/9, 11π/9, and 16π/9. When *τ* approaches zero, Equation (10) then reduces to

(12) $$\varphi_{\mathrm{sq}}=-\arcsin\left(\frac{U}{36I_{0}J_{1}(\phi_{m})}\right).$$

### 2.3. Control of the Modulation Amplitude

According to Equation (9), the optimum *signal-to-noise ratio* (*SNR*) for *U* can be obtained when *J*_1_(*ϕ*_m_) takes the maximum value, which means that *ϕ*_m_ ≈ 1.84 and *J*_1_(*ϕ*_m_) ≈ 0.58. Define that

(13) $$Κ=(I(\pi /2)+I(3\pi /2))/2=I_{0}+I_{0}\cos(\varphi_{\mathrm{sq}})\cos(\phi_{m}),$$

(14) $$S=(I(3\pi /2)-I(\pi /2))/2=I_{0}\sin(\varphi_{\mathrm{sq}})\sin(\phi_{m}),$$

(15) $$A=(I(0)+I(\pi ))/2=I_{0}+I_{0}\cos(\varphi_{\mathrm{sq}}),$$

(16) $$B=(I(\pi /6)+I(7\pi /6))/2=I_{0}+I_{0}\cos(\varphi_{\mathrm{sq}})\cos\left(\phi_{m}/2\right),$$

(17) $$C=(I(\pi /3)+I(4\pi /3))/2=I_{0}+I_{0}\cos(\varphi_{\mathrm{sq}})\cos(\sqrt{3}\phi_{m}/2).$$

The dynamic range of the open-loop gyro is (−π/2, π/2), and thus cos(*φ*_sq_) > 0. Based on Equations (15)–(17), we can determine that

(18) $$M=\frac{A-B}{A-C}=\frac{1-\cos\left(\phi_{m}/2\right)}{1-\cos\left(\sqrt{3}\phi_{m}/2\right)}.$$

Equation (18) suggests that if the driving voltage of PZT is controlled to make |*M* − 0.385| ≤ *err*_1_ (*err*_1_ is a predefined acceptable control error for PZT, which is 0.0003 in this work), *ϕ*_m_ could be controlled to (1.84 ± 0.005) rad.

### 2.4. Control of Optical Intensity

According to the demodulation algorithm defined by Equation (9), the averaged optical intensity *I*_0_ should be settled to a predefined constant value (i.e., the first derivative equals zero) in the demodulation control process. Consequently, the gyro can realize a stable output as long as the *I*_0_ is closed-loop controlled. Since the SNR of the FOG mainly depends on the shot noise that is proportional to √*I*_0_ [18], the gyro’s input optical intensity should be increased as much as possible in order to obtain more accurate demodulation results. However, a higher optical intensity of the light source leads to its reduced lifetime and increased relative intensity noise [19] and thus decreased SNR. Therefore, considering both factors, in this work, a moderate optical intensity *I*_0_ corresponding to an optical power of 10 μW is chosen for our gyro. The control process is then given as follows. With *ϕ*_m_ = 1.84 rad, substitute *ϕ*_m_ into Equations (13) and (16), and thus,

(19) $$I_{0}=(0.606K+0.266B)/0.872.$$

The control of optical power can be achieved by changing the drive current of the light source to make |*I*_0_ − *T*| ≤ *err*_2_ (*err*_2_ is the predetermined acceptable optical power control error depending on the control accuracy of the SLED, which is 0.005 μW in this work), where *T* is the target value of the optical power, i.e., 10 μW in this work.

### 2.5. Suppression of the Bias with a Periodic Flip of Modulation

The rotation speed of the gyro can be derived from Equation (12). However, in the actual implementation process, crosstalk between circuit signals will also cause an error that is independent of the rotation speed and manifested as the zero-bias. Stable zero-bias can be calibrated and removed in a system application [20]. However, this kind of error is often non-stable in most cases, greatly affecting the accuracy of a positioning system.

The traditional approach uses elaborate circuit layouts to isolate and shield all the noise sources [21]. However, this approach requires a larger circuit layout area, which is unsuitable for developing a small-volume open-loop fiber-optic gyroscope. It has been found that most noise results from the gyro’s electric source and ground wire [22], which is independent of the modulation polarity. According to Equation (12), because *J*_1_(*ϕ*_m_) is an odd function, the error can be easily eliminated by flipping the modulation polarity regularly and changing the output polarity simultaneously. The detailed modulation process is as follows.

**Figure 4:**
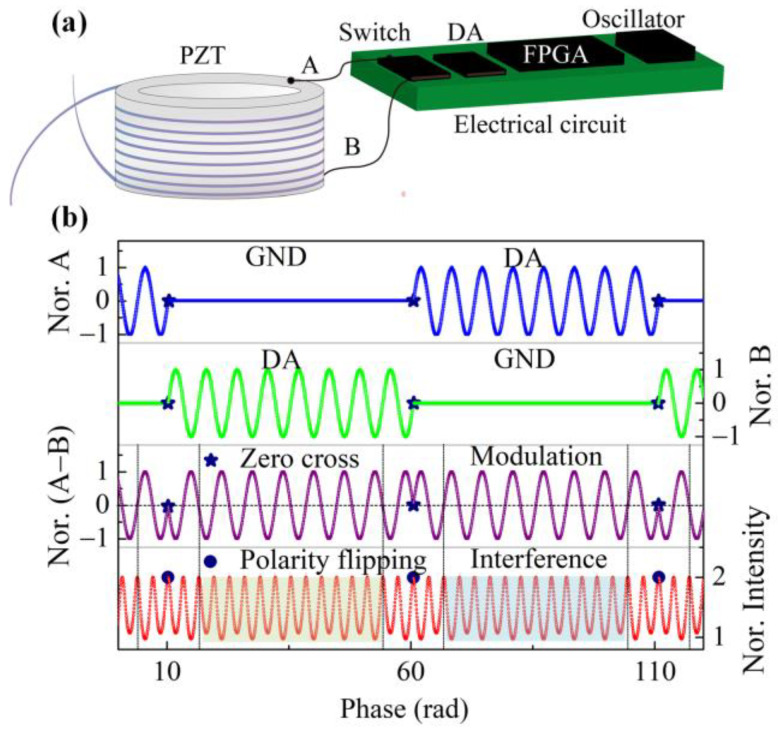
(**a**) Diagram of PZT control; (**b**) periodically flipped driving pattern, the modulation generated by the driving, and the corresponding interference signal of the gyro (assuming that the gyro has a Sagnac phase shift of 0.05 rad for clarity).

The sinusoidal driving waveform of PZT is generated from DA controlled by the FPGA whose clocking is supplied by a crystal oscillator shown in Figure 4a. The exact moment of zero-crossing can be adjusted accurately, which corresponds to the extreme point of the interference signal. The driving voltage applied to the PZT modulator across points A and B is switched periodically between the DA output and the ground. As shown in Figure 4b, each modulation segment lasts for eight full modulation cycles, and the phase of consecutive modulation segments is increased by π, meaning that the modulation polarity of PZT is flipped periodically at the zero-crossing point. This form of modulation pattern not only realizes the modulation inversion but also ensures the continuity of the interference waveform. Data from one cycle each before and after the flipping are discarded during the demodulation process to prevent gyro noise caused by PZT flipping. Considering the bias due to crosstalk, the output of the FOG takes the form of

(20) $$\begin{array}{l} \varphi_{\mathrm{sq}}(\phi_{m})=-\arcsin\left(\frac{U}{36I_{0}J_{1}(\phi_{m})}\right)+\varepsilon \\ \varphi_{\mathrm{sq}}(-\phi_{m})=\arcsin\left(\frac{U}{36I_{0}J_{1}(\phi_{m})}\right)+\varepsilon \end{array},$$

where *φ*_sq_(*ϕ*_m_) and *φ*_sq_(−*ϕ*_m_) are the demodulated phases with positive and negative modulation, respectively, and *ε* is the error due to electric crosstalk. Hence, the bias due to the crosstalk can be eliminated, and the accurate gyro output can be achieved by synchronously flipping the polarity of the gyro’s output according to

(21) $$\varphi_{\mathrm{sq}}=\frac{\varphi_{\mathrm{sq}}(-\phi_{m})-\varphi_{\mathrm{sq}}(\phi_{m})}{2}.$$

## 3. Software, Hardware Implementation, and System Construction

Based on the analysis above, the proposed algorithm uses simple arithmetic operations to replace the traditionally used and more complex multi-frequency convolution algorithm, thereby reducing the requirements for the computing power of the processor. Most open-loop FOGs with digital control [23] use both an FPGA and DSP for signal processing, whereby the FPGA is used to control the sampling timing, and the DSP is used for complex calculation.

In our design, a low-cost Xilinx Spartan-7 FPGA is sufficient for the implementation of all the algorithms. The whole electrical circuit is about half the cost of conventional open-loop FOG products and with a compact footprint of only 35 × 22 mm^2^. The hardware diagram and software process are shown in Figure 4. The FPGA controls the analog-to-digital converter (ADC) to sample the interference light and the digital-to-analog converter (DAC) to achieve the closed-loop feedback of the SLED output power and the phase of the PZT modulator. The computation required by the digital demodulation of the Sagnac phase with bias suppression is also conducted in the FPGA. The timing of the above processes is precisely controlled with a crystal oscillator. The initial values of the control parameters as a function of start-up temperature are determined during the calibration process after the gyro is assembled. They are tabulated and pre-stored in the memory of the gyro’s circuit board for the fast retrieval of multiple control parameters at the start-up within the full temperature range [24].

**Figure 5:**
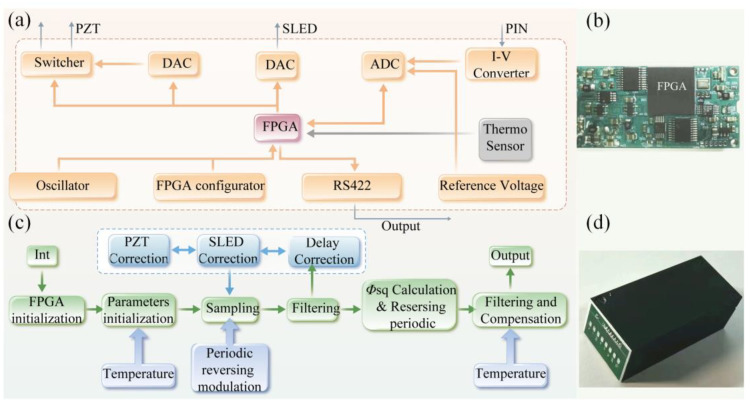
(**a**) Circuit hardware diagram, (**b**) picture of the circuit hardware, (**c**) software process of FOG, and (**d**) picture of the FOG.

A prototype open-loop FOG, based on the schematic shown in Figure 1, was assembled with the circuit hardware and software configuration as discussed above. An ultra-fine 40 μm polarization-maintaining optical fiber of 200 m long was wound on a ring skeleton with a size of 35 × 18 × 22 mm^3^ using a quadrupole symmetrical winding method. An in-house packaged miniaturized GaAs SLED light source of a center wavelength of 830 nm was adopted. It was mounted on a heat sink that had thermal contact with the mechanical structure of our open-loop FOG, and no active cooling was applied to it. The output power of the light source can be more than 500 μW under a driving current of 60 mA with a fiber-coupled power of 50 μW. The 2 couplers were in-house developed fused taper-type couplers using the 40 μm polarization-maintaining fiber. The beam-splitting ratio error was less than 5%, and the polarization-maintaining capability was greater than 20 dB. The polarizer was also an in-house developed 45° tiled fiber Bragg grating whose extinct ratio is more than 35 dB [25,26]. A 100 kHz sinusoidal-modulated PZT phase modulator with about 1 m long wrapped fiber was integrated into the system to reduce the size and cost of the FOG. All the optical components and streamlined circuit board with our new algorithm fit into a volume of only 25 × 20 × 40 mm^3^ to construct the miniaturized open-loop fiber-optic gyroscope, which is shown in Figure 5d.

## 4. Determinations of Gyroscope Performance

Fast start-up is an important metric for a fiber-optic gyro used in the field of control and automation. Prior to gyro performance testing, it is essential to evaluate the start-up process of the control parameters for the new algorithm.

**Figure 6:**
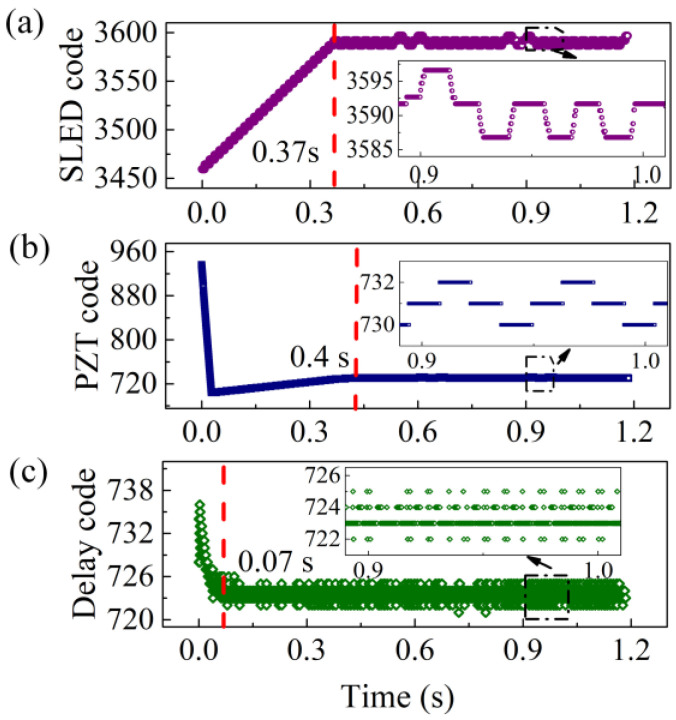
Dynamic control results of (**a**) SLED, (**b**) PZT, and (**c**) sampling delay during the startup process.

The experimental results show that our proposed control algorithm can control all objectives, including the power of the light source, the PZT modulation phase, and the sampling delay within 0.4 s to reach their steady state, as shown in Figure 6. This means that taking into account the 0.6 s required for FPGA initialization, the gyro start-up time only adds up to less than 1 s.

**Figure 7:**
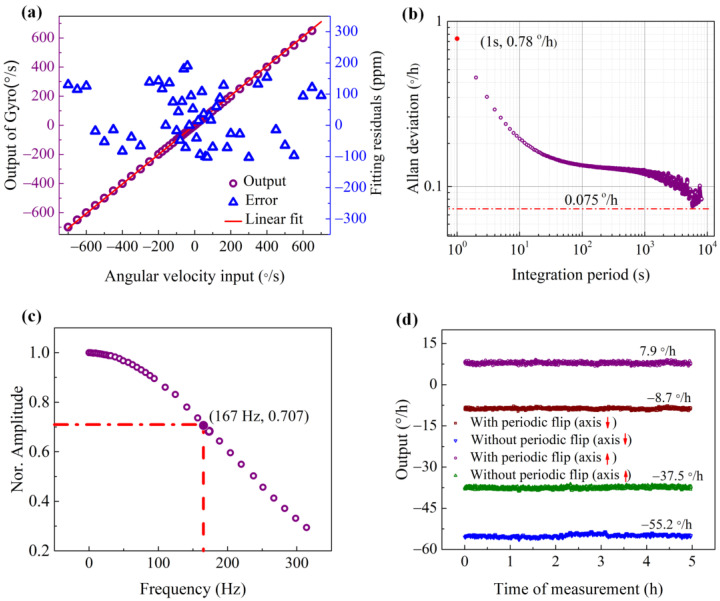
The measured results for our digital open-loop fiber-optic gyroscope. (**a**) Gyro output at different angular rates, (**b**) results for Allan variance testing, (**c**) gyro amplitude-frequency characteristics, and (**d**) bias comparison with the different algorithms.

The performance of this open-loop FOG prototype is characterized according to the *IEEE 952-2008 protocol*. The test of our FOG was conducted in a Class-10k cleanroom with a temperature of 25 ± 1 °C, a humidity of 40 ± 5%, a magnetic field of less than 1 Gauss, and an air pressure of 1 atm. The gyroscope was mounted on a fixed base attached to a separate foundation to isolate the external vibration. The output of this FOG was close to linear with the angular rotation of the test platform on which the FOG was mounted. Figure 7a shows that the scale factor error was less than 200 ppm. Allan variance was used to evaluate the static characteristics of the gyro, i.e., the *ARW* for white noise and bias instability (*B*_I_) [27]. As shown in Figure 7b, on the Allan variance curve, *ARW* can be estimated by dividing the value at 1 s by 60 to be 0.012°/√h and *B*_I_ can be calculated by dividing the bottom of the curve by 0.664 to be 0.11°/h. To verify the dynamic performance of the gyro, we tested the amplitude–frequency characteristics of the gyroscope with a swing table under different swing frequencies [28]. The FOG had excellent dynamic characteristics with a 3 dB bandwidth of 160 Hz, as shown in Figure 7c, which can meet the application requirements in the field of automatic driving, robot control, etc. [29]. The Gaussian-like decay of the amplitude–frequency curve indicates that the bandwidth of the gyro was mainly limited by the low-pass filter in the algorithm, which can be further improved by optimizing the filter. The effectiveness of the bias elimination algorithm was also verified, as shown in Figure 7d, and after turning on the periodic flip, the gyro’s bias decreases from −55.2°/h to −8.7°/h, which is close to the celestial component of the earth’s rotation speed of −8.4 °/h at the test location (Xi’an, Shaanxi Province, China, with its latitude of 34° N). We placed the gyro in reverse (the direction of the sensitive axis changed from pointing to the ground to the sky) and repeated the above experiment. The output of the gyro changed from −37.5°/h to 7.9°/h after turning on the periodic flip. The earth’s rotation speed was eliminated by adding the forward and reverse output together and dividing this sum by two. By periodically flipping its sensitive axis, the gyro gives an output of −0.25°/h, compared with −46.4°/h obtained without periodic flip.

According to our experimental results, the open-loop fiber-optic gyro with our new control and demodulation algorithm achieves very good performance. However, our algorithm can be further improved by optimizing its low-pass filter to boost the gyro’s 3 dB bandwidth and squeezing more sampling points within a single cycle to reduce gyro noise and suppress nonlinear errors. Currently, 85% of the resource of the low-cost FPGA used in this work has been utilized. The optimization of our above algorithm will possibly have to overhaul the entire structure of the algorithm as well as the implementation of its key functionality to save considerable resources for arithmetic operation.

## 5. Conclusions

In this work, a new algorithm for an open-loop fiber-optic gyroscope has been proposed. Unlike the traditional resource-intensive synchronous demodulation of multiple harmonic components, our method relies on only a few discrete points on the sinusoidal modulation curve. Based on our algorithm, stable control and angular velocity demodulation of the gyro were both realized. In addition, a periodic flipping modulator driving pattern was proposed and successfully demonstrated to suppress the bias due to the electric crosstalk. Our algorithm greatly reduces the requirements for processing power and simplifies the gyro circuit. As a result, the cost and the volume of the gyro can be greatly reduced. Our prototype digital FOG has a volume of only 25 × 20 × 40 mm^3^, and the comprehensive testing reveals its bias instability of less than 0.15°/h, an *ARW* of less than 0.015°/√h, a start-up time of less than 1 s, and a 3 dB bandwidth beyond 160 Hz. We believe that this kind of performance can meet the navigation needs of autonomous driving vehicles [30] and the control and automation needs of many other applications such as androids.

## Data Availability

The data presented in this study are available on request from the corresponding author.
